# Laparoscopic adrenalectomy: preoperative data, surgical technique and clinical outcomes

**DOI:** 10.1186/s12893-018-0456-6

**Published:** 2019-04-24

**Authors:** Giuseppe Di Buono, Salvatore Buscemi, Attilio Ignazio Lo Monte, Girolamo Geraci, Vincenzo Sorce, Roberto Citarrella, Eliana Gulotta, Vincenzo Davide Palumbo, Salvatore Fazzotta, Leonardo Gulotta, Domenico Albano, Massimo Galia, Giorgio Romano, Antonino Agrusa

**Affiliations:** 1 Department of Surgical, Oncological and Oral Sciences (Di.Chir.On.S.), University of Palermo, Italy, Via L. Giuffrè, 5, 90127 Palermo, Italy; 2 Department of Experimental Biomedicine and Clinical Neurosciences, University of Palermo, Palermo, Italy; 3 Department of Radiology, University of Palermo, Palermo, Italy

**Keywords:** Laparoscopic adrenalectomy, Laparoscopic surgery, Adrenalectomy, Endocrine surgery, Cushing syndrome, Adrenal Incidentaloma

## Abstract

**Background:**

laparoscopic adrenalectomy has become the standard treatment for adrenal lesions. The better clinical outcoms of laparoscopic technique are valid for treatment of small benign masses (< 5–6 cm), instead there are still open questions in literature regarding the correct management of larger lesions (> 6 cm) or in case of potentially malignant adrenal tumors. The aim of this study is to evaluate the outcomes of laparoscopic adrenalectomy in a referral surgical department for endocrine surgery.

**Methods:**

at the University Hospital Policlinico “P. Giaccone” of Palermo between January 2010 and December 2017 we performed a total of 81 laparoscopic adrenalectomy. We created a retrospective database with analysis of patients data, morphologic and hormonal characteristics of adrenal lesions, surgical procedures and postoperative results with histological diagnosis and complications.

**Results:**

Mean size of adrenal neoplasm was 7,5 cm (range 1.5 to 18 cm). The mean operative time was 145 min (range 75–240). In statistical analysis lenght of surgery was correlated to the lesion diameter (*p* < 0.05) but not with pre-operative features or histological results. 5 intraoperative complications occurred. Among these patients 4 presented bleeding and 1 a diaphagmatic lesion. No conversion to open surgery was necessary and no intraoperative blood transfusion were required. Mean estimated blood loss was 95 ml (range 50–350). There was no capsular disruption during adrenal dissection. Mean length of hospital stay was 3.7 days (range 3–6 days).

**Conclusions:**

Laparoscopic adrenalectomy is a safe procedure with low rate of morbidity. An accurate preoperative radiological examination is fundamental to obtain a stringent patients selection. The lesion diameter is related to longer operative time and appeares as the main predictive parameter of intraoperative complications but these results are not statistically significant. On the other side secreting adrenal tumors require more attention in operative management without increased rate of postoperative complications.

## Background

Since first description in 1992 by Gagner laparoscopic adrenalectomy (LA) has become the standard treatment for adrenal lesions [1]. Over the years several studies have identified the advantages of laparoscopic technique with reduction in morbidity and perioperative mortality due to less operative blood loss, lower complication rates, less postoperative pain, shorter hospital stay and better cosmetic results when compared with open adrenalectomy (OA) [2]. On the basis of positive outcomes of LA performed with traditional lateral trans-peritoneal approach, other surgical techniques have also been developed as posterior retroperitoneoscopic adrenalectomy (PRA) or robotic adrenalectomy (RA) [3–5] These excellent clinical achievements are valid for treatment of small benign masses (< 5–6 cm), instead there are still open questions in literature regarding the correct management of larger lesions (> 6 cm) or in case of potentially malignant adrenal tumors [6]. In these patients increases the risk of incomplete resection, capsular disruption, local recurrence and peritoneal dissemination [7].With the enlarged diffusion of laparoscopy many surgeons carry out LA even if they have poor experience with adrenal pathologies. On the basis of these considerations, the aim of this study is to evaluate the outcomes of LA in a referral surgical department for endocrine surgery.

## Methods

In our Department of General and Emergency Surgery at the University Hospital Policlinico “P. Giaccone” of Palermo between January 2010 and December 2017 we performed a total of 81 LA. We created a retrospective database with analysis of patients demographic data, previous abdominal surgery, American Society of Anesthesiologists (ASA) score, morphologic and hormonal characteristics of adrenal lesions, surgical procedures, postoperative results, histological diagnosis and in particular cases also mid- and long-term follow-up. Only patients with certain primary elements of malignant adrenal neoplasm on radiological examination like local invasion and/or distant metastases were excluded from the study because underwent to OA [8]. All surgical procedures were performed by the same surgical team experienced in endocrine and laparoscopic surgery in order to obtain a standard technique.

### Preoperative evaluation

Before surgery all patients underwent a complete preoperative checkup including cardiological counseling for evaluating general performance status and endocrine study with complete hormonal tests to identify functioning adrenal tumors. Today there is no consensus on the optimal diagnostic approach to the incidentally discovered adrenal masses. For the diagnosis of Cushing’s syndrome we carried out urine free cortisol test, serum cortisol and ACTH at 8 a.m. and 6 p.m. (ACTH drawn into an EDTA tube, cooled). We also used dexamethasone suppression test with evaluation of cortisol secretion suppression after oral administration of 1 mg dexamethasone at 11–12 p.m. The cortisol serum concentration was measured on the following day in the morning after overnight fasting (at 8–9 a.m.). In case of aldosteronoma we dosed aldosterone serum concentration and plasma renin activity (PRA) in upright position as indirect indicator of renin secretion. In our experience we did not perform adrenal venous sampling to discern between unilateral and bilateral aldosterone overproduction because we had no cases of bilateral lesions. We examined the adrenal medullary function with dosage of fractionated urinary metanephrines and catecholamines using High Performance Liquid Chromatography (HPLC) technique. With the same HPLC we tested dehydroepiandrosterone (DHEA) and dehydroepiandrosterone sulphate (DHEA-S) in patients with hyperandrogenism. In order to obtain blood pressure stabilization a pharmacological preoperative treatment was done by alpha-1 blocker or calcic inhibitors in case of pheocromocytoma (PCC); we used spironolactone and potassium for Conn’s syndrome. In all cases we administered antithrombotic and antibiotic prophylaxis. We carried out routinely an abdominal contrast-enhanced Computed tomography (CT) scan to study the morphological characteristics and size of the lesion. Abdominal magnetic resonance imaging (MRI) was used in selected patients with unclear features to the CT scan. We analyzed the presence of fat components within the lesions through frequency selective fat suppression and chemical shift suppression or differences in cellularity on T_1_ and T_2_ weighted sequences [8]. We used functional imaging as [^75^se]cholesterol, [^131^I]metaiodobenzylguanidine scintigraphy or [^18^F]fluorodeoxyglucose positron emission tomography (FDG-PET) only if necessary such as in case of bilateral secreting lesions. A diagnosis of adrenal cortical carcinoma (ACC) was based on the patients history (e.g. hormonal symptoms with virilizing tumor) and radiologic findings. We excluded from this study patients with imaging features of malignancy as local infiltration of the surrounding structures, venous invasion or systemic metastasis. In these cases we performed OA. In addiction we also excluded from the study a right LA for large bleeding angiomyolipoma performed in urgent setting. The mere size of the lesion was not considered a signal of malignant lesion [9]. Indication for LA included hormone secreting tumors (Cushing’s lesion, Conn’s syndrome, pheochromocytomas) and all non-functioning tumors larger than 4 cm.

### Surgical procedure

LA was carried out with a transperitoneal approach with the patient in the lateral decubitus position with an inclination of 50–60° relative to the operating table which was broken to extend the space between the last rib and the iliac crest [10]. We always used Veress needle to induce pneumoperitoneum also in case of previous abdominal surgery. For right adrenalectomy we positioned four trocars in the right subcostal region. The right lobe of the liver was mobilized by division of triangular and coronary ligament to expose the adrenal loggia. Then the posterior peritoneum overlying the right adrenal gland, in Albarran-Chatelin space, was dissected using Harmonic scalpel™ (Ethicon Endo Surgery INC - Johnson & Johnson, NJ, USA). The tissue dissection began along the lateral border of the inferior vena cava: we used right renal vein as anatomic landmark and then we identified a short adrenal vein that was clipped and divided. After vascular ligation we reached the ileopsoas muscle plane and the adrenal gland was mobilized until it was completely free. For left-side adrenal resection we positioned three trocars in the left subcostal region. The procedure began with adequate mobilization of splenic flexure of the colon and then we divided splenocolic and splenophrenic ligament to medially mobilize the splenic-pancreatic block. On the contrary of the right side where we had a well defined Albarran-Chatelin space, for the left side the landmarks were more difficult to identify. We could consider a quadrilater between spleen and pancreatic tail, upper pole of the kidney, left renal vein and ileo-psoas muscle. The peritoneal dissection was done from up-to-down until the left renal vein was reached and from this we identified adrenal vein that was clipped and divided. In some cases we used the diaphragmatic vein as landmarks. Completed the vascular dissection the resection proceeded as previously described for the right adrenal gland on the posterior muscular plane from upper pole of the kidney to the diaphragm with the aim to empty the adrenal loggia. On the left side we always used Tisseel™ (Baxter International Inc. - Deerfield, Illinois, USA) for repositioning splenic-pancreatic block in order to avoid wandering spleen. At the end of the procedure surgical specimens were positioned in endo-bag and removed through the operative trocar or in case of large lesions with sovrapubic mini-laparotomy to improve cosmesis and prevent incisional hernia [11, 12]. The total operative time, the partial surgical times (exposure of the adrenal loggia, identification of the adrenal vein, dissection time) and intraoperative complications were noted respectively in the operative report and in the video recordings of the procedure.

### Postoperative management

Postoperative pharmacological management changed according to the hormonal diagnosis of the adrenal mass. In all patients normal diet and mobilization started on the first postoperative day. We positioned abdominal drainage only in large adrenal masses and difficult surgical procedures. In these cases drainage was removed on the first or second postoperative day. Postoperative complications, defined as an unexpected postoperative course, were graded according to the Dindo-Clavien classification [13]. For statistical analysis we only considered postoperative medical or surgical complications with grade equal or superior to 2.

### Statistical analysis

Statistical analysis were performed with SPSS 20.0 (SPSS Inc., Arlington, Virginia). Continuous variable was expressed as the mean. Categorical Data were compared with Chi-square analysis and continous data with Student *t* test. A *p* value < 0.05 was considered statistically significant.

## Results

Between January 2010 and December 2017, 81 patients (34 male and 47 female) with a mean age of 55.4 years (range 38–81) underwent to LA, 50 on the right side and 31 on the left side. We did not treat bilateral lesions. Patients and tumors characteristics are summarized in Table 1. We registered that the major part of patients (73 cases) had ASA score 3 with several comorbidities. In 23 cases (28.4%) we found previous abdominal or retroperitoneal surgery including 9 (11.1%) appendectomies, 10 (12,3%) gynecological procedure and 4 (4.9%) different surgeries (2 laparoscopic cholecystectomies, 1 open right hemicolectomy for cancer and 1 open surgical procedure for renal stones) [14]. Indications for LA included 18 (22.2%) Cushing’s syndrome, 10 (12.3%) Conn’s syndrome, 14 (17.3%) pheochromocytomas, 1 (1.2%) adrenal metastasis, 2 (2.5%) adrenocortical carcinomas (ACC), 17 (21%) non-secreting adenomas, 11 (13.6%) myelolipomas, 3 (3.7%) adrenal cysts, 4 (4.9%) hemangiomas and 1 (1.2%) nodular hyperplasia [15]. Histological examination showed inside the Cushing’s syndrome 2 adrenal cortical carcinomas. Inside the 14 pheochromocytomas 2 were malignant. We also found a cystic pheochromocytoma with histological signs of malignancy in a patients with preoperative diagnosis of adrenal cyst. The lesion diagnosed as metastasis in patient with history of endometrial cancer was really primary ACC (Table 2).

**Table 1:** Demographic characteristcs of patients and indications for surgery

	Mean; *n* (%)
Sex (M/F)	34/47 (42%/58%)
Age	55.4 (range 38–81)
ASA score	
1	–
2	5 (6.2%)
3	73 (90.1%)
4	3 (3.7%)
Previous abdominal surgery	
- appendectomies	9 (11.1%)
- gynecological surgery	10 (12.3%)
- other procedures	4 (5%)
Pre-operative diagnosis	
- Cushing’s syndrome	18 (22.2%)
- Pheochromocytoma	14 (17.3%)
- Conn’s syndrome	10 (12.3%)
- Non-secreting adenoma	17 (21%)
- Myelolipoma	11 (13.6%)
- Adrenal cyst	3 (3.7%)
- Hemangioma	4 (4.9%)
- Nodular Hyperplasia	1 (1.2%)
- Adrenocortical carcinoma	2 (2.5%)
- Metastasis	1 (1.2%)

**Table 2:** Histological diagnosis

	*n* (%)
Cortisol-producing adenoma	16 (19.7%)
Pheochromocytoma	12 (14.8%)
Aldosterone-producing adenoma	10 (12.5%)
Non-functional adenoma	17 (21%)
Myelolipoma	11 (13.6%)
Adrenal cyst	2 (2.5%)
Hemangioma	4 (4.9%)
Nodular Hyperplasia	1 (1.2%)
Adrenocortical carcinoma	5 (6.2%)
Malignant Pheochromocytoma	3 (3.7%)
Metastasis	–

Details of procedure are reported in Table 3. Mean size of adrenal neoplasm was 7,5 cm (range 1.5 to 18 cm). Aldosteronoma was associated with the smallest size (1.5 cm) while myelolipoma was the largest observed mass (18 cm). The mean operative time was 145 min (range 75–240). In statistical analysis lenght of surgery was correlated to the lesion diameter (*p* < 0.05) but not with pre-operative features or histological results. With evaluation of the video recordings of the surgical procedures we also took into consideration the partial operative times: exposure of the adrenal loggia, identification of the adrenal vein and dissection time. The results showed that in the functioning masses and in particular in pheochromocytomas was greater the time of identification of the adrenal vein. In the large adrenal masses (> 6 cm) all three partial times were increased but above all the adequate exposure of the adrenal loggia. 5 (6.2%) intraoperative complications occurred. Among these patients 4 presented bleeding: 1 vena cava injury treated with intracorporeal sutures and positioning of pre-rolled TachoSil®; 1 from a small hepatic accessory vein that was isolated and clipped; 1 caused from a spleen injury during left adrenalectomy, in this case hemostasis was obtained with use of hemostatic matrix (Floseal®); 1 from peri-renal tissue solved by positioning of Hemopatch®. In another patient with a large right adrenal masses (14 cm) we observed a diaphagmatic lesion treated with suture and intra-corporeal knotting with no transthoracic drainage [16, 17]. No conversion to open surgery was necessary and no intraoperative blood transfusion were required. Mean estimated blood loss was 95 ml (range 50–350). Hemodynamic change with intraoperative hypertension occurred in 2 cases during pheochromocitomas removal without particular complications. There was no capsular disruption during adrenal dissection. There was none postoperative death. According to the Dindo-Clavien classification [13] for statistical analysis we only considered postoperative medical or surgical complications with grade equal or superior to 2. We excluded 4 cases of sovrapubic wound infections open at the patient bedside (Grade 1). In one case we observed wound hematoma that required postoperative blood transfusion but not surgical intervention (Grade 2). In 3 patients was necessary prolonged antibiotic intake after hospital discharge due to localized pneumonia (Grade 2) None patient underwent a reoperation due to complications (Grade 3). In the statistical analysis there was no significant correlation between mean tumor size, histological type, intraoperative complication and postoperative outcome (*p* value > 0.05). Only mean operative time resulted significant longer in large adrenal tumors (> 6 cm). Mean length of hospital stay was 3.7 days (range 3–6 days). Mean follow up was 38.5 months (range 3–72 months). All patients with malignant pheochromocytoma and adrenal cortical cancer were still alive after mean follow-up of 26,2 months (range 2–70). No local recurrence and port-side metastasis was noted.

**Table 3:** Details of surgical procedures and postoperative complications

	Mean; *n* (%)
Adrenalectomy	
- Right	50 (61.7%)
- Left	31 (38.3%)
- Bilateral	–
Operative time	145 min (range 75–240)
Lesion diameter	7.5 cm (range 1.5–18)
Conversion to open surgery	–
Intraoperative complication	
- Bleeding	3 (3.7%)
- Intraoperative blood transfusion	–
- Organ lesion	2 (2.5%)
Postoperative complication	
- Blood transfusion	1 (1.2%)
- Respiratory disease	3 (3.7%)

## Discussion

Since 1992 LA has become the standard treatment in patients with small benign adrenal masses [1]. When comparing with open technique, LA offered better clinical outcomes, reduced surgical trauma, lower perioperative morbidity and mortality, shorten hospitalization and better cosmetic results [2]. In order to reduce surgical trauma and improvement clinical postoperative outcome several authors developed posterior retroperitoneoscopic adrenalectomy (PRA). A randomized clinical trial by Barczynski et al. [18] showed excellent results of both techniques, anterior and posterior, in unilateral small tumors with statistically significant advantage of PRA in operative time, blood loss and postoperative course. However, the retroperitoneal approach reduced its benefits in the case of larger adrenal masses, so this approach could be preferred for bilateral small lesions and for patients with multiple past abdominal surgery that increased the risk of intraoperative complications and rate of conversion to the open surgery [19]. Laparoscopic surgery was also more difficult to learn than open surgery because required different psychomotor skills. Robotic adrenalectomy (RA) presented multiple advantages with three-dimensional vision and increased degrees of freedom of the surgical instruments. A recent meta-analysis [20] that included 798 patients compared operative parameters and clinical outcomes between RA and LA. The authors concluded that RA was a safe and feasible technique with reduced blood loss and shorter hospital stay than LA. Laparoscopic approach seemed to be a more rapid technique when comparing to RA. On the other hand, some authors considered the use of 3D laparoscopic technology with a traditional transperitoneal approach so as to combine the advantages of a standardized and diffused surgical procedure with an improved vision [21]. These authors analyzed LA for particular deep location of adrenal loggia with theoretical maximum advantage of a 3D system. The results showed the better visualization in depth perception with effect on surgical precision but without significant differences in term of operative time and intraoperative complications [22, 23]. In our study we registered pre-operative data like ASA score, radiological features and hormonal tests. In literature age and ASA score were direct related to increased of length of hospital stay and postoperative complications [24]. Radiological features and tumor size appeared as a very important predictive parameter of outcome. The results showed the large adrenal masses (> 6 cm) were associated with duration of surgery and with an increased risk of intraoperative incident. Many author reported lesion diameter over 5–6 cm as independent predictive factors for conversion [25, 26]. Functioning masses and in particular pheochromocytomas required longer operative time to identify the adrenal vein but these differences were no statistically significant. From analysis of partial operative time we also demonstrated that in large adrenal masses significantly increased the time necessary to approach the adrenal loggia. First exposure of adrenal gland by section of triangular and coronary liver ligament on the right side and splenocolic and splenophrenic ligament on the left side must be performed carefully in order not to damage diaphragm, spleen and pancreatic tail. Bleeding was the main intraoperative complications and injuries of the vena cava, renal vein or hepatic vein represented a real dangerous situation with increased conversion rate [27].We did not report conversion to open surgery. In case of intraoperative complications we performed a conservative laparoscopic management of vascular, splenic and diaphragmatic injuries. The conversion instead was mandatory in case of intraoperative evidences of malignancy like periadrenal tissue infiltration or vascular invasion. We considered capsular disruption as an intraoperative complication too. Literature data suggested that the main limitation during laparoscopic dissection for large and potentially malign adrenal tumors was incomplete resection and capsular disruption with increased risk of local recurrence and intra-abdominal neoplastic dissemination [28], but the tumor size per se could not be considered as an absolute controindication to LA. A correct preoperative patients selection and a meticulous surgical technique could limit that risk. Postoperative complication rate was 5% with one blood transfusion and three respiratory diseases. According to Dindo-Clavien scale rate we considered medical or surgical complications with grade ≥ 2 for statistical analysis with no significant correlation between mean tumor size, histological type and postoperative outcome (*p* value > 0.05). Coste T. et al. [24] reported that postoperative medical complications were mainly respiratory diseases and various infections.

## Conclusion

In this study we used retrospective data like demographic, radiological and hormonal characteristics and prospective parameters such as partial time of surgical procedures and evaluation of intraoperative complications obtain from recordings video of LA. The study period was limited from January 2010 to December 2017 and the all procedures were performed in a single center by an experienced endocrine and laparoscopic surgical team. The limitations were related to lower rate of postoperative complication with not significant results in terms of association between preoperative characteristics and postoperative outcomes. Nevertheless, it is possible to confirm that LA is a safe procedure with low rate of morbidity. An accurate preoperative radiological examination is fundamental to obtain a stringent patients selection. The lesion diameter is related to longer operative time and appeares as the main predictive parameter of intraoperative complications but these results are not statistically significant. On the other side secreting adrenal tumors require more attention in operative management without increased rate of postoperative complications.

## Abbreviations

ACC: Adrenal Cortical Carcinoma
BP: Blood Pressure
LA: Laparoscopic Adrenalectomy
OA: Open Adrenalectomy
PCC: Pheochromocytoma
PRA: Posterior Retroperitoneoscopic Adrenalectomy
RA: Robotic Adrenalectomy
